# Histopathologic differences between human T-lymphotropic virus type 1 (HTLV-1)-positive and HTLV-1-negative polymyositis

**DOI:** 10.1186/1742-4690-8-S1-A39

**Published:** 2011-06-06

**Authors:** Hazem M Abdullah, Itsuro Higuchi, Ryuji Kubota, Eiji Matsuura, Akihiro Hashiguchi, Nashwa H Abdelbary, Yukie Inamori, Hiroshi Takashima, Shuji Izumo

**Affiliations:** 1 Division of Molecular Pathology, Center for Chronic Viral Diseases,Kagoshima University, Kagoshima, Kagoshima, 890-8544, Japan; 2 Department of Human Pathology, Faculty of Medicine, Mansoura University, 60 El, Mansoura, Dakahlia governorate, 35516, Egypt; 3 Department of Neurology and Geriatrics, Graduate School of Medical and Dental Sciences, Kagoshima University, Kagoshima, Kagoshima 890-8520, Japan

## Objectives

Epidemiological studies show that human T-lymphotropic virus type 1 (HTLV-1) is closely associated with polymyositis (PM). However, the pathogenic roles of HTLV-1 in PM remain unknown. This study aims to assess skeletal muscle morphology in the presence of HTLV-1 infection to compare the histopathological findings of HTLV-1-positive and HTLV-1-negative PM.

## Methods

Among the 68 patients with inflammatory myopathy diagnosed through muscle biopsy over the previous 10 years at Kagoshima University Hospital, we retrospectively selected 21 patients with PM not associated with any other disease; we evaluated HTLV-1 positivity through serology, confirmed it by nested PCR using DNA extracted from muscles, and then assessed the tissue viral load. Meticulous histopathological examination was performed using routine histochemical and immunohistochemical staining, and specimens from selected cases were examined by electron microscopy.

## Results

The clinical and histopathological findings of muscle biopsy specimens of HTLV-1-positive (n = 11) and HTLV-1-negative PM cases (n = 10) were compared. Compared with HTLV-1-negative patients, HTLV-1-positive patients exhibited protracted clinical courses, prominent endomysial infiltrates, infrequent necrotic fibers, and prominent regenerative activities. Moreover, they showed frequent cytochrome c oxidase deficiency and ultrastructural abnormalities in mitochondria.

## Conclusions

These differences are significant but not specific to HTLV-1-positive PM. Therefore, HTLV-1 may induce the clinical and histopathological modifications of PM observed in this study.

